# Impacts of soil salinity on Bt protein concentration in square of transgenic Bt cotton

**DOI:** 10.1371/journal.pone.0207013

**Published:** 2018-11-07

**Authors:** Yong-Hui Wang, Jin Gao, Ming-Fa Sun, Jian-Ping Chen, Xiang Zhang, Yuan Chen, De-Hua Chen

**Affiliations:** 1 Institute of Agricultural Sciences of Jiangsu Coastal Area, Observation and Experimental Station of Saline Land of Costal Area, Ministry of Agriculture, Yancheng, P. R. China; 2 Key Laboratory of Crop Genetics and Physiology of Jiangsu Province, Yangzhou University, Yangzhou, P. R. China; Institut Sophia Agrobiotech, FRANCE

## Abstract

Insect-resistance of transgenic *Bacillus thuringiensis* (Bt) cotton varies among plants organs and with different environmental conditions. The objective of this study was to examine the influence of soil salinity on Bt protein concentration in cotton squares and to elucidate the potential mechanism of Bt efficacy reduction. Two cotton cultivars (NuCOTN 33B and CCRI 07, salt-sensitive and salt-tolerant) were subjected to salinity stress under four natural saline levels in field conditions in 2015 and 2016 and seven regimes of soil salinity ranged from 0.5 to 18.8 dS m^-1^ in greenhouse conditions in 2017. Results of field studies revealed that Bt protein content was not significantly changed at 7.13 dS m^-1^ salinity, but exhibited a significant drop at the 10.41 and 14.16 dS m^-1^ salinity. The greenhouse experiments further showed similar trends that significant declines of the insecticidal protein contents in squares were detected when soil salinity exceeded 9.1 dS m^-1^. Meanwhile, high salinity resulted in significant reduction in contents of soluble protein and total nitrogen, activities of nitrate reductase (NR), glutamine synthetase (GS) and glutamic-pyruvic transaminase (GPT), but increased amino acid content, activities of protease and peptidase in cotton squares. High salinity also decreased root vigor (RV), root total absorption area (RTA) and root active absorption area (RAA). The extent of decrease of Bt protein content was more pronounced in NuCOTN 33B than CCRI 07, and CCRI07 exhibited stronger enzymes activities involved in square protein synthesis and higher levels of RV, RTA and RAA. Therefore, the results of our present study indicated that insecticidal protein expression in cotton squares were significantly affected by higher salinity (equal to or higher than 9.1 dS m^-1^), reduced protein synthesis and increased protein degradation in squares and reduced metabolic activities in roots might lead to the decrease of Bt protein content in squares.

## Introduction

Since the first transgenic *Cry1Ac Bacillus thuringiensis* (Bt) cotton variety was commercialized in 1997 in China, more than 3.7 million hectares of transgenic Bt cottons are cultivated in China, accounting for 96% of the total cotton-growing area [1]. Application of Bt cotton increased economic income, decreased environmental pollution and enhanced safety protection for workers by minimizing pesticide use [2,3]. Unfortunately, resistance of Bt cotton to lepidopteran pests is unstable in field applications [4,5]. Loss of insect-resistance was closely associated with reduced level of insecticidal proteins [6,7], which was affected by cotton genotype [8], plant age [9], plant organ [10], gene type and insertion site [11,12], and environmental determinants [13,14].

Salinity is one of the most important environmental factors affecting production and stability of *Cry1Ac* protein in transgenic Bt cotton. Some studies have demonstrated that NaCl stress decreased Bt protein level and affected the efficiency to control bollworms in controlled conditions such as greenhouse and laboratory [15–17]. Other studies also have shown a negative relationship between Bt protein content and soil salinity in field conditions [18]. However, previous studies usually focused on the inhibition effects of salt stress on Bt protein expression in cotton leaves, reports about the variation of Bt protein content in cotton squares under soil salinity was rare. Compared with leaves, square as the primary target of cotton bollworms, usually showed lower insecticidal protein content than leaves and should be more vulnerable [7,10]. Therefore, to accurately assess the effects of salinity on insecticidal efficiency of Bt cottons, investigations of Bt protein level in cotton squares under salinity stress should be performed.

There were a few reports regarding underlying mechanisms of reduction of insecticidal ability in Bt cottons under salinity stress. It has been reported that decreased soluble protein and total nitrogen content under salinity stress impaired expression of Bt protein in cotton leaves [15,17]. Some studies have found that the content of insecticidal toxin in cotton leaves was related to the contents of total nitrogen, soluble protein and amino acid, as well as enzyme activities involved in protein synthesis and degradation, such as nitrate reductase (NR), glutamine synthetase (GS), glutamic-pyruvic transaminase (GPT), protease and peptidase [19–21]. However, little is known how nitrogen physiological metabolism varies in cotton squares under soil salinity. Moreover, the expression of *Cry1Ac* gene also affected by nitrogen metabolism in Bt cotton [22]. Therefore, we hypothesized that high salinity might also affected insecticidal toxin contents in cottons squares through regulating N metabolism.

Roots play a major role in plants because they directly contact with the soil, absorb and transport essential nutrients and water from the soil [23–24]. Capacity of nitrogen acquisition by roots affects nutrition status and plants growth [25–27]. Thus, Harmful effects of high salinity on roots might subsequently affect N uptake and then reduced protein synthesis including Bt protein in Bt cotton. However, no research was conducted to examine the relationship between Bt protein expression and root physiological processes such as roots vigor and root absorption area. Thus, to better understand the underlying mechanisms of variation of Bt protein content, changes of root absorption ability in response to soil salinity gradients should be investigated.

In this study, two transgenic Bt cotton cultivars with different salt sensitivity were subjected to different regimes of soil salinity under field and greenhouse conditions to determine the characteristics of insecticidal protein expression in cotton squares in response to different soil salinity levels and explore potential mechanisms of Bt protein content reduction in squares under salinity stress.

## Materials and methods

### Ethics statement

No specific permit is required for the experiments on Bt cottons in P. R. China. Field experiments were conducted at the Cotton Experimental Station of Jinhai Farm (120°49′E, 33°59´N, Dafeng District, Jiangsu Province, China), which is our own experimental field. Experiments in this area do not need any approval. Field studies did not involve endangered or protected species.

### Field experiments

Two batches of field experiments were conducted in 2015 and 2016. Soil salinity was measured, and then four areas with similar texture and nutrient level but with different salinities (1.06, 7.13, 10.41 and 14.16 dS m^-1^) were selected (Table 1). Each area was divided into blocks (4.8 m × 15 m) and randomly used for cotton planting.

**Table 1:** Environmental parameters of soil from four field testing areas. TN: total nitrogen; AN: available nitrogen; AP: available phosphorus; AK: available potassium; BD: bulk density; ECe: electrical conductivity. Data were measured using surface soil (0 to 20 cm) in early spring before transplanting seedlings in 2015.

Salinity level	pH	TN	AN	AP	AK	BD
ECe (ds·m^-1^)	(H_2_O)	(g kg^-1^)	(mg kg^-1^)	(mg kg^-1^)	(mg kg^-1^)	(g cm^-3^)
1.06	8.01	0.98	82.4	16.7	232.4	1.23
7.13	8.10	1.01	81.7	15.4	224.8	1.21
10.41	8.05	0.97	83.6	17.1	235.2	1.19
14.46	8.04	0.95	80.5	16.3	233.6	1.20

Two Bt transgenic cotton cultivars with different salt tolerance, were employed in the present study [28,29]. One cultivar, NuCOTN 33B (salt-sensitive), was obtained from Jiangsu Academy of Agricultural Sciences (Nanjing, China), and the other cultivar, CCRI 07 (salt-tolerant), was bred by the China Cotton Research Institute (Anyang, China). Seeds of these two cultivars were sowed in a nursery bed in April 2016 and April 2017. When three true leaves were developed, healthy and similar-sized seedlings were transplanted to field blocks with spacing of 0.80 m × 0.28 m. During cotton growth, fertilization, insect and weed control followed the local management.

Fifteen days after appearance of the first bud, 15 cotton squares were collected from each block. Samples were immediately frozen in liquid nitrogen, and then stored at -40°C for determination of Bt protein content.

### Greenhouse experiments

A semi-open greenhouse was constructed at the Agricultural Experimental Station of Yancheng Academy of Agricultural Sciences (33°25′N, 120°12′E, Yancheng, Jiangsu Province, China). Walls of the greenhouse can be removed to allow air circulation. The top plastic cover was transparent, allowing sunlight and avoiding precipitation.

Clay loam soil was collected from top soil (0 to 30 cm) in the experimental station. After air-dried, soils were sieved through a 100-mesh net. Sodium carbonate (77.7%), magnesium chloride (7.3%), magnesium sulfate (9.6%), calcium chloride (3.3%), and potassium chloride (2.1%) were mixed as salty reagent. Six salinity gradients were prepared, in which salty reagent was mixed in soil as ratio of 0.1%, 0.2%, 0.3%, 0.4%, 0.5% and 0.6%. Pure soil without addition of salty reagent was used as control. Electrical conductivity of a saturated-paste extract (ECe) in control and treatments was measured equal to 0.5 (control), 2.6, 5.9, 9.1, 12.3, 15.5 and 18.8 dS m^-1^, respectively.

Seedlings of NuCOTN 33B and CCRI 07 were bred in a nursery bed till formation of three true leaves, and then transplanted into pots (45 cm diameter, 37 cm deep) which were filled with 25 kg of mixed soil. Each pot included one seedling and each treatment was repeated for 20 times. For each pot, 8.7 g of N (using urea; 30% at transplanting stage, 40% at early flowering stage and 30% at peak flowering stage), 4.3 g of P_2_O_5_ (using triple super phosphate; 50% at transplanting stage and 50% at early flowering stage) and 5.8 g of K_2_O (using potassium sulfate, 50% at transplanting stage and 50% at early flowering stage) were fertilized.

Thirty squares growing on the first to third fruiting branches were collected per treatment, at 15 days after appearance of bud. Fifteen squares were frozen with liquid nitrogen and then stored at -40°C for protein analyses, and the left 15 squares were dried at 80°C to constant weight and then stored in a glass dryer at room temperature for determination of total nitrogen content.

At the end of experiments, four individuals were cut at the cotyledonary node for each treatment. Roots were dug out and washed with water to determine root vigor, total and active absorption areas of root systems.

### Determination of *Cry1Ac* protein level

Protein level of *Cry1Ac* was determined using the ELISA method [30]. Approximately 0.3 g of square tissues were homogenized in 1 ml of extraction buffer (Na_2_CO_3_ 1.33 g, DTT 0.192 g, NaCl 1.461 g, Vc 0.5 g, dissolved in 250 ml distilled water) and then completely transferred to 5 ml centrifuge tube by washing homogenizer with 2 ml of buffer. After shaken by hand and stored at 4°C for 4 hours, samples were centrifuged at 10 k × g at 4°C for 20 min, and supernatant was filtered through a C18 Sep-Pak Cartridge (Waters, Milford, MA) before ELISA determination. ELISA analysis of *Cry1Ac* was performed using a commercial kit (Scientific Service, Inc., Beijing) following the manufacture’s protocol. Briefly, microtitration plates were coated with standard *Cry1A* insecticidal protein or samples by incubation at 37°C for 4 hours. *Cry1A* antibody was developed as described by Weiler et al. [31] and then added into each well. After incubation at 37°C for 30 min, solution was discarded and horseradish peroxidase-labeled goat anti-rabbit secondary antibody was added, incubated at 37°C for 30 min and then discarded. Finally, the buffered enzyme substrate (orthopenylenediamino) was added and incubated in dark at 37°C for 15 min. The reaction was terminated using 3 M H_2_SO_4_. Absorbance at 490 nm of each well was determined. The results were calculated following Weiler et al. [31].

### Determination of root activity and absorption area

Root activity was measured using the triphenyltetrazolium chloride (TTC) method [32]. Total and active absorption areas of fresh roots were determined using the methylene blue dye method [33].

### Determination of total nitrogen content

Squares were completely digested using H_2_SO_4_ and H_2_O_2_ and then content of total nitrogen content was measured following Kjeldahl’s method [34].

### Determination of free amino acid and soluble protein contents

Square tissues (0.3 g) were homogenized in 5 ml of cold phosphate buffer (50 mM KH_2_PO_4_, pH 7). After centrifugation at 12 k × g for 15 min, supernatant was collected. Content of total free amino acid content was determined using the ninhydrin method [35] and expressed as mg of amino acid per gram of fresh weight. Glycine solution was used to prepare standard curve. Content of total soluble protein was determined using brilliant blue G-250 regent [36] and bovine serum albumin (BSA) was used to prepare standard curve.

### Determination of enzyme activities

Nitrate reductase (NR) activity was determined according to Ding et al. [37] with slight modification. Square tissue (0.3 g) was homogenized in 4 ml of 0.1 M phosphate buffer (pH 7.5) and then centrifuged at 12 k × g for 20 min at 4°C. Reaction solution including 0.4 ml of nicotinamide adenine dinucleotide (NADH), 1.2 ml of 0.1 M KNO_3_ and 0.4 ml of extraction solution was maintained at 25°C for 30 min. Afterwards, 1 ml of sulphanilamide was added to stop reaction. 0.1 M sodium phosphate (pH 7.5) was added instead of NADH as control. Next, 1 ml of 1% N-1-naphthylethylenediamine dihydrochloride was added to form red color and incubated for 15 min before centrifugation at 12 k × g for 10 min. Absorbance at 540 nm was measured to calculated NR activity. Sodium nitrite nitrogen was applied for standard curve.

To determine glutamine synthetase (GS) activity, samples were extracted according to Ding et al. [37] and then assayed according to Oaks et al. [38]. Briefly, sample (0.5 g) was homogenized using a chilled mortar in 3 ml of 5 mM sodium phosphate buffer (pH 7.2) containing 50 mM Na_2_SO_4_ and 0.5 mM Na_2_-EDTA. After centrifugation at 20 k × g for 20 min at 4°C, 1.2 ml supernatant was reacted with 0.3 ml of 0.3 M Na-Glu, 0.6 ml of 0.25 M imidazole-HCl (pH 7.0), 0.2 ml of 0.5 M MgSO_4_, and 0.4 ml of 0.03 M adenosine triphosphate (ATP) at 25°C for 5 min. Next, 0.2 ml of 1.0 M hydroxylamine was added and further incubated at 25°C for 20 min. Reaction was terminated by adding 0.8 ml of mixed reagent (10% FeCl_3_·6H_2_O, 50% HCl and 24% trichloroacetic acid. After 20 min, mixtures were centrifuged at 5 k × g for 10 min and absorbance at 540 nm was measured. GS activity was calculated based on standard curve using c-glutamyl-hydroxamate.

Activity of glutamic-pyruvic transaminase (GPT) was assayed as described by [39]. Square sample (0.3 g) was homogenized in 5 ml of 0.05 mM Tris-HCl (pH 7.2) and centrifuged at 26 k × g for 10 min at 4°C. 0.2 ml of supernatant was mixed with 0.5 ml of 0.8 M alanine (prepared in 0.1 M Tris-HCl, pH 7.5), 0.2 ml of 0.1 M 2-oxoglutarate solution and 0.1 ml of 2 mM pyriodoxal phosphate solution, and then incubated at 37°C for 10 min. Next 0.1 ml of trichloroacetic acid solution was added to stop reaction. Absorbance of solution at 520 nm was measured. GPT activity was calculated based on authentic pyruvate standards [40].

To determine peptidase and protease activities, samples were extracted as described by Carrasco and Carbonell [41]. Peptidase activity was analyzed according to Setlow [42] with slight modification. 0.1 ml of extracted supernatant was mixed with 1 ml of buffer containing 50 mM Tris-HCl (pH 8.0), 1 mM MnCl_2_ and 5 mM peptide and then incubated at 37°C for 30 min. Reaction was terminated by adding of 1 ml of 1% ninhydrin solution (1 mg/ml cadmium acetate, 85% ethanol and 15% acetic acid). Absorbance at 505 nm was measured. Protease activity was determined using azocasein as substrate. Reaction mixture contained 0.4 ml of 10 mg ml^-1^ azocasein, 0.4 ml of 0.05 M succinate buffer (pH 5.5) with 10 mM mercaptoethanol, and 0.2 ml of extracted supernatant. After incubation at 35°C for 1 h, 1 ml of 1 N perchloric acid was added to stop reaction. Reaction mixture was centrifuged at 8 k × g for 15 min and absorbance at 400 nm was recorded [43].

### Data analyses

Statistical data analyses were conducted using SPSS 11.0 (SPSS Software Inc., USA). Homogeneity of all data was checked using the Levene’s test. Afterwards, one-way analysis of variance (ANOVA) was performed to test effects of salinity on each parameter, followed by Duncan’s multiple range tests.

## Results

### Changes of insecticidal protein level in cotton square

Results of field study revealed that salinity stress affected level of insecticidal protein expression in squares. Along with increasing salinity, Bt protein content declined in squares of both cultivars (Table 2). Compared with treatment with 1.06 dS m^-1^, Bt protein content did not change in treatment with 7.13 dS m^-1^, but significantly decreased in treatments with higher salinity.

**Table 2:** Impacts of salinity on Bt protein content in cotton square (ng g^-1^ FW) of two Bt cultivars in field study during 2015 and 2016 (n = 4). Different letters represent significant differences in the same column (Duncan multiple range tests, P < 0.05).

Salinity level	2015		2016	
ECe (ds m^-1^)	NuCOTN 33B	CCRI 07	NuCOTN 33B	CCRI 07
1.06	567.3±9.3^a^	541.4±17.5^a^	573.2±14.4^a^	553.6±21.0^a^
7.13	553.2±24.6^a^	558.3±12.9^a^	558.1±6.5^a^	542.9±14.4^a^
10.41	483.6±13.8^b^	479.6±6.1^b^	504.5±11.3^b^	504.7±10.7^b^
14.46	407.9±21.2^c^	441.8±13.8^c^	451.4±15.7^c^	471.2±13.8^c^

**Fig 1:**
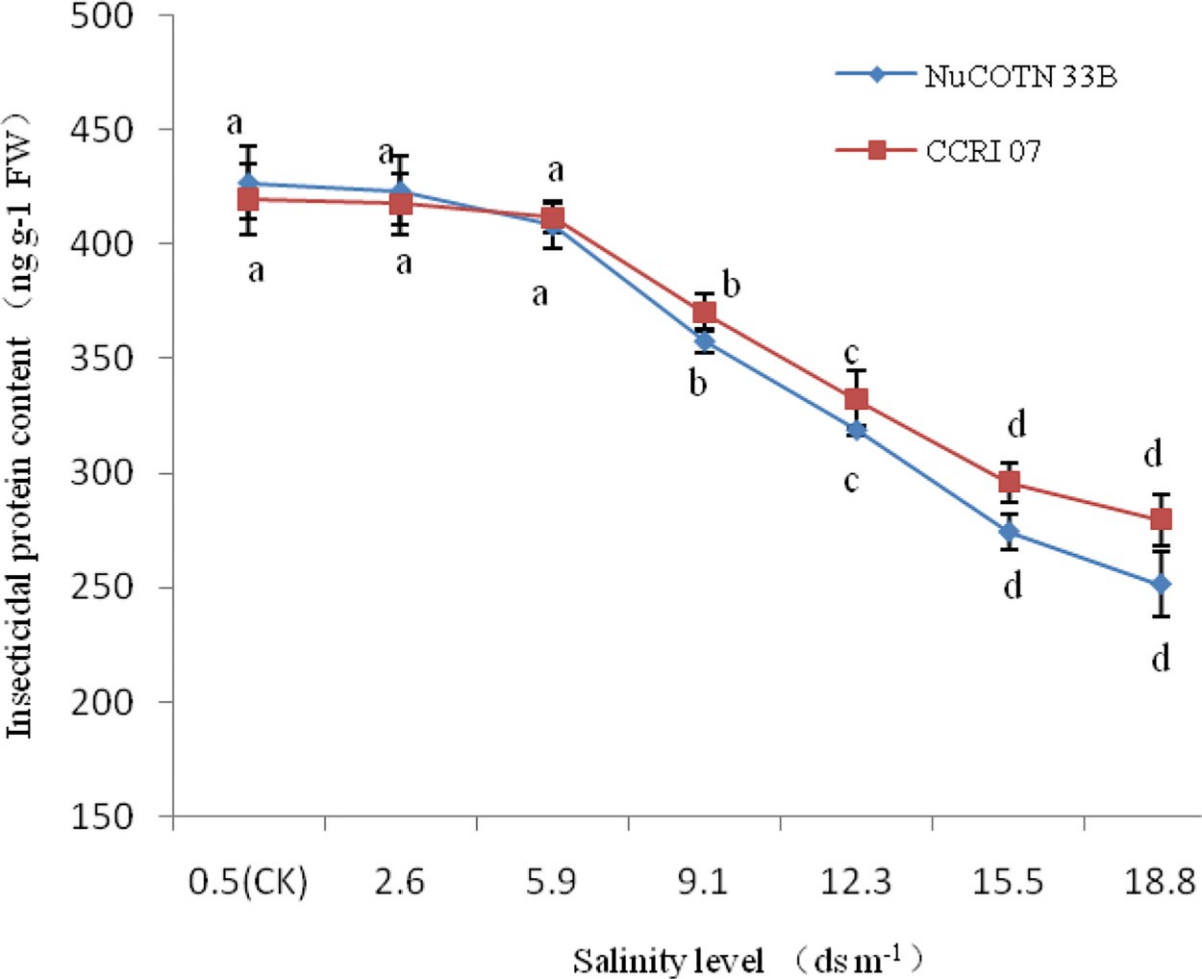
Effects of soil salinity on insecticidal protein content in square of two cotton cultivars (mean ± SE, n = 4). Different letters represent significant differences (Duncan multiple range test, *P* < 0.05).

Greenhouse experiments also showed a similar result that high salinity decreased Bt protein content in squares when (Fig 1). No significant difference was detected when the salinity level ranged from 0.5 to 5.9 dS m^-1^. Significant reductions were detected when the salinity levels went up to 9.1 dS m^-1^, compared to the control. Furthermore, the magnitude of decrease was greater in the cultivar NuCOTN 33B compared to CCRI 07, especially at 15.5 or 18.8 dS m^-1^ salinity levels.

### Nitrogen metabolism in cotton square

Square nitrogen metabolism related parameters were negatively affected by salinity treatments. Total nitrogen content and soluble protein content decreased with increasing soil salinity. There was little change when the salinity levels ranged from 0.5 to 5.9 ds m^-1^ (Table 3). With increasing salinity levels from 9.1 to 18.8 ds m^-1^, the content of total nitrogen and soluble protein in squares was reduced significantly and the degree of inhibition was greater in NuCOTN 33B, than in CCRI 07. In contrast, free amino acid concentrations increased with the increment of soil salinity level from 0.5 ds m^-1^ to 18.8 ds m^-1^, and significant difference for both cultivars was investigated when salinity was above 9.1 ds m^-1^.

**Table 3:** Impacts of salinity on total nitrogen, soluble protein and amino acid contents in squares of two Bt cotton cultivars under greenhouse conditions in 2017 (n = 4). Different letters represent significant differences in the same column (Duncan multiple range tests, P < 0.05).

Salinity level	Total nitrogen content (%)		Soluble protein content (mg g^-1^ FW)		Amino acid content (μmol g^-1^ FW)	
ECe (ds m^-1^)	NuCOTN 33B	CCRI 07	NuCOTN 33B	CCRI 07	NuCOTN 33B	CCRI 07
0.5	3.46±0.05^a^	3.63±0.07^a^	1.62±0.03^a^	1.78±0.06^a^	22.9±0.4^e^	25.0±0.5^d^
2.6	3.52±0.08^a^	3.64±0.03^a^	1.70±0.12^a^	1.77±0.04^a^	22.6±0.2^e^	25.2±0.5^d^
5.9	3.31±0.14^a^	3.55±0.11^a^	1.52±0.08^a^	1.70±0.02^a^	22.3±1.2^e^	24.8±0.6^d^
9.1	3.05±0.04^b^	3.29±0.06^b^	1.24±0.08^b^	1.55±0.07^b^	26.5±0.6^d^	27.3±0.3^c^
12.3	2.78±0.11^c^	3.02±0.03^b^	1.06±0.05^c^	1.42±0.12^bc^	28.7±1.3^c^	32.4±1.1^b^
15.5	2.35±0.08^d^	2.61±0.06^c^	0.82±0.11^d^	1.23±0.09^c^	31.1±0.6^b^	33.1±0.7^b^
18.8	2.21±0.07^d^	2.58±0.04^c^	0.71±0.05^d^	1.12±0.04^c^	33.6±1.1^a^	35.1±0.4^a^

Compared with the control, slight reductions have been detected in the enzymes activities of NR, GS and GPT when the salinity levels increased from 2.6 to 9.1 ds m^-1^, while their activities began to decrease significantly at higher salinity levels (Table 4). The decrease of the activities of GS and GPT were greater than NR. Moreover, enzyme activities of GS and GPT were significantly lower in NuCOTN 33B compared to CCRI 07 under salinity conditions (Table 4).

**Table 4:** Impacts of salinity gradient on nitrate reductase (NR), glutamine synthetase (GS) and glutamic-pyruvic transaminase (GPT) activities in squares of two Bt cotton cultivars under greenhouse conditions in 2017 (n = 4). Different letters represent significant differences in the same column (Duncan multiple range tests, P < 0.05).

Salinity level	NR activity (μg g^-1^ FW h^-1^)		GS activity (A mg^-1^ pro h^-1^)		GPT activity (μmol g^-1^ FW h^-1^)	
ECe (ds m^-1^)	NuCOTN 33B	CCRI 07	NuCOTN 33B	CCRI 07	NuCOTN 33B	CCRI 07
0.5	4.20±0.03^a^	4.27±0.06^a^	0.728±0.011^a^	0.843±0.023^a^	15.03±0.32^a^	17.08±0.31^a^
2.6	4.18±0.08^a^	4.34±0.12^a^	0.704±0.016^ab^	0.823±0.017^a^	15.23±0.48^a^	17.22±0.55^a^
5.9	4.16±0.04^a^	4.17±0.05^a^	0.688±0.023^b^	0.742±0.016^b^	14.49±0.46^a^	16.38±0.58^a^
9.1	3.86±0.08^b^	3.81±0.09^b^	0.635±0.018^c^	0.708±0.011^b^	12.28±0.37^b^	13.24±0.44^b^
12.3	3.63±0.06^bc^	3.72±0.04^b^	0.583±0.030^d^	0.643±0.026^c^	12.07±0.40^b^	13.02±0.29^b^
15.5	3.55±0.06^c^	3.59±0.12^bc^	0.405±0.022^e^	0.499±0.019^d^	9.86±0.13^c^	11.66±0.54^c^
18.8	2.85±0.14^d^	3.25±0.13^c^	0.356±0.014^f^	0.481±0.025^d^	8.19±0.60^d^	10.10±0.32^d^

Changes of protease and peptidase activities were similar to content of amino acid. No significant differences were detected in square protease and peptidase activities among the salinity levels from 0.5 to 5.9 ds m^-1^. Compared with the control, protease and peptidase activities in cotton squares significantly increased for both cultivars in treatments with salinity equal to or higher than 9.1 ds m^-1^ (Table 5). Although the value of protease and peptidase activities was lower in NuCOTN 33B than that of CCRI 07 at the same salinity level, the increase percent was relatively high in NuCOTN 33B at all salinity regimes compared to the control.

**Table 5:** Impacts of salinity on protease and peptidase activities in squares of two Bt cotton cultivars under greenhouse conditions in 2017 (n = 4). Different letters represent significant differences in the same column (Duncan multiple range tests, P < 0.05).

Salinity level	Protease activity (μg g^-1^ FW h^-1^)		Peptidase activity (μmol g^-1^ FW h^-1^)	
ECe (ds m^-1^)	NuCOTN 33B	CCRI 07	NuCOTN 33B	CCRI 07
0.5	3.42±0.14^e^	3.57±0.18^d^	0.67±0.04^d^	0.83±0.05^c^
2.6	3.28±0.17^e^	3.34±0.14^d^	0.71±0.03^d^	0.90±0.03^c^
5.9	3.56±0.17^e^	3.77±0.08^d^	0.65±0.02^d^	0.87±0.05^c^
9.1	3.93±0.08^d^	3.95±0.06^c^	0.76±0.02^c^	0.97±0.07^b^
12.3	4.15±0.07^c^	4.32±0.10^b^	0.89±0.05^b^	1.04±0.05^b^
15.5	4.65±0.11^b^	4.43±0.17^b^	0.87±0.04^b^	1.13±0.06^ab^
18.8	4.91±0.07^a^	4.72±0.11^a^	0.99±0.07^a^	1.20±0.06^a^

### Physiological characterization of root

Statistical analyses indicated that soil salinity significantly influenced RV, RTA and RAA. Compared with the control, treatments with 2.6 and 5.9 dS m^-1^ did not significantly change RV, RTA and RAA of both cultivars. However, when salinity level increased to 9.1 dS m^-1^ or higher, RV, RTA and RAA in both cultivars was significantly reduced (Table 6). Moreover, RV, RTA and RAA were relatively lower in NuCOTN 33B than those of CCRI 07.

**Table 6:** Impacts of salinity on root vigor (RV), root total absorption area (RTA) and root active absorption area (RAA) of two Bt cultivars under greenhouse conditions in 2017 (n = 4). Different letters represent significant differences in the same column (Duncan multiple range tests, P < 0.05).

Salinity level	RV (ug g^-1^FW h^-1^)		RTA (m^2^)		RAA (m^2^)	
ECe (ds m^-1^)	NuCOTN 33B	CCRI 07	NuCOTN 33B	CCRI 07	NuCOTN 33B	CCRI 07
0.5	307.6±6.7^a^	321.3±14.3^a^	4.23±0.13^ab^	4.48±0.17^ab^	2.01±0.07^a^	2.34±0.10^a^
2.6	310.2±15.9^a^	328.6±12.6^a^	4.63±0.28^a^	4.72±0.09^a^	2.26±0.09^a^	2.53±0.14^a^
5.9	278.6±17.5^ab^	301.4±9.8^a^	3.99±0.14^b^	4.24±0.13^b^	1.83±0.09^b^	2.25±0.15^a^
9.1	255.4±20.6^b^	268.9±5.7^b^	3.47±0.08^c^	3.88±0.07^c^	1.65±0.12^b^	1.93±0.08^b^
12.3	223.4±11.7^b^	237.8±12.5^c^	3.07±0.11^d^	3.54±0.16^d^	1.41±0.04^c^	1.72±0.13^bc^
15.5	157.1±8.0^c^	184.3±13.1^d^	2.76±0.14^e^	3.37±0.15^de^	1.14±0.11^d^	1.46±0.14^c^
18.8	105.3±16.3^d^	159.7±18.5^d^	2.37±0.03^f^	2.92±0.23^e^	0.92±0.14^d^	1.23±0.06^c^

### Correlation analyses

Correlation analyses showed that Bt protein content in squares was significant positive correlation with RV, RTA, RAA, total nitrogen content, soluble protein content and activities of NR, GS and GPT, but negative correlation with amino acid content, activities of protease and peptidase (Tables 7 and 8).

**Table 7:** Correlation coefficients between physiological indexes and Bt protein content in cotton squares under soil salinity stress in 2017.

Cultivars	Total N	Soluble protein content	NR activities	GS activities	GPT activities	Free amino acid	Protease activities	Peptidase activities
NuCOTN 33B	0.970**	0.969**	0.923**	0.953**	0.930**	-0.948**	-0.944**	-0.877**
CCRI 07	0.969**	0.955**	0.916**	0.939**	0.941**	-0.939**	-0.870**	-0.852**

n = 21

** and * represent significance at the 1% and 5% level, respectively.

**Table 8:** Correlation coefficients between roots physiological indexes and Bt protein content under soil salinity stress in 2017.

Cultivars	RV	RTA	RAA
NuCOTN 33B	0.956**	0.956**	0.952**
CCRI 07	0.957**	0.919**	0.928**

n = 21

** and * represent significance at the 1% and 5% level, respectively.

## Discussion

It has been reported that soil salinity affected plant growth, nutrition and reduce insect resistance and Bt protein content in cotton leaves [19,23,44]. However, the response of Bt protein content to abiotic stress varied among different cotton organs [45,46]. In our present study, a preliminary experiment was conducted in field-grown cotton in saline land with four salinity gradient and indicated that high soil salinity caused a significant Bt toxin decrement in cotton squares. The results in controlled environment further confirmed the results and suggested that the salinity level below 9.1 dS m^-1^ had little effect on the squares Bt protein concentration, but significant reduction of Bt protein efficacy was noted when soil salinity exceeded 9.1 dS m^-1^. Our result was consistent with the findings of Iqbal et al. [16], who reported a negative relationship between the Bt protein content and NaCl concentration, and salt level above 10 dS m^-1^ decrease the *Cry1Ac* toxin level significantly at different cotton growth stages. Thus, we concluded that the reduced insect-resistance of cotton square was also closely correlated to salinity levels, and 9.1 dS m^-1^ might be the soil salinity threshold affecting squares Bt protein concentration. The present research indicated that salt-induced Bt protein content decline could be related with changes in protein content, enzymes activities and root vitality, as discussed below.

Maintenance of stabilized protein synthesis is very critical for normal functions of cells under high salinity stress because of their involvement in most metabolic processes [47–49]. Bt protein as a proportion of soluble proteins, was affected inevitably by the abilities of protein metabolism in cotton squares [17].NR, GS and GPT are major enzymes participating in protein synthesis. NR reduces nitrate (NO_3_^−^) to nitrite (NO_2_^−^), which is rate limiting enzyme in production of protein. GS catalyzes the combination of glutamate and ammonia (NH_4_^+^) to form glutamine and organic amino acids. GPT catalyzes the transfer of an amino group from L-alanine to α-ketoglutarate and produces pyruvate and L-glutamate [50]. In this study, we observed a non-significant reduction in activities of NR, GS and GPT in square of both cultivars at lower soil salinity. In contrast, a significant decrease was detected at 9.1 ds m^-1^ salinity levels or higher. The reduction of enzymes activities under salinity conditions might be a result of low enzymes synthesis, or direct activity suppression. The contents of soluble protein and total N also showed a similar change trend with increasing soil salinity. In greenhouse experiment, Bt protein concentration showed significant positive correlation with NR, GS and GPT activities. Thus, these results suggested the inhibition of activities of NR, GS and GPT limited squares protein synthesis in Bt cotton exposed to salinity stress, as shown by lower Bt protein content in cotton squares. These findings agree with the previous studies of the hypothesis on the inducement of insect-resistant reduction [20,21,51].

Salt stress always induced accumulation of free amino acid in plant tissues. However, there are two different views on the interpretation of phenomenon. Several studies have suggested plants synthesize higher amino acids content in cells under abiotic stress because amino acids serve as organic solutes and energy, and could improve osmotic adjustment and mitigate salinity stress induced cellular damages [52,53]. By contrast, other reporters have documented that an increase in amino acid content originates from strengthened proteolysis [22,54]. Our present work supported the latter speculation, since protease and peptidase activities also enhanced sharply when salinity levels exceeded 9.1 ds m^-1^. In addition, reduced soluble protein content observed under high salinity conditions further supported this point. Li et al. [19] have also reported that enhanced protease activity was accompanied with decreased protein content in cotton boll under water deficit stress. Thus, these results indicated that protein degradation might be another reason for the decrement of square Bt protein concentration. However, whether protein degradation is a dominate factor or not should be further investigated.

Many researches have demonstrated that salt stress adversely affected morphology and function of cotton roots such as root length, root intensity, root absorption and root redox activity [55,56]. In this study, it was found that RV, RAA and RTA reduced with increased soil salinity levels, and decreased markedly when salinity level was above 9.1 dS m^-1^. High salinity decreased photosynthesis of plants and inhibited carbohydrate metabolism, resulting in decreased dry matter accumulation rate and reduced biomass allocation to root correspondingly [57,58]. Furthermore, salt stress also induced oxidative damage caused by over production of reactive oxygen species (ROS) in plant roots, led to protein degradation and metabolic disorders in plant tissues [59,60]. Thus, the reduction of RV, RAA and RTA was partly due to excessive salt in soil which was attributed to poor root development. Chen et al. [56] reported that high salinity disturbed nutrient balance in cotton plants, more N was accumulated in the leaves and stem parts rather than reproductive organs. Some studies have suggested that nitrate reductase and glutamine synthetase belong to induced enzymes, and their activities were elicited by NO_3_^-^ and NH_4_^+^ accumulation in plant cells [50]. Hence, the reduction of NR and GS activities in our study might also be relevant to inadequate N supply due to diminished root metabolic activity caused by salinity stress. The correlation analysis further showed that there was a significant positive correlation between RV, RAA, RTA and Bt protein content for both cultivars. Therefore, we could conclude that restrictions in absorption and transportation of nitrogen nutrition from roots led to nitrogen deficiency in squares, ultimately resulting in lower nitrogen strength and lower Bt protein content.

Plants with different salt sensitivity display diverse levels of adaption against salinity. These adaptions were partly correlated with the ability of absorption and assimilation of nitrogen [48,50,61]. Our results were consistent with results of preceding studies [17] that Bt protein content in salt-sensitive cultivar NuCOTN 33B was more affected by salinity stress than salt-tolerant cultivar CCRI 07, although marked drop occurred at salinity level of 9.1 ds m^-1^ for both cultivars. Under salt stress, CCRI 07 maintained a higher soluble protein content and total N content than NuCOTN 33B compared with the control. This was partly due to the relatively stronger N absorption ability of CCRI 07as indicated by RV, RTA and RAA. Although high salinity reduced the activities NR, GS and GPT, and elevated protease and peptidase significantly for both cultivars, the magnitude of variation was larger for NuCOTN 33B than CCRI 07. In consistency with our results, Li et al. [62] also found higher NR and GS activities in Bt cultivars with high salt tolerance compared to salt sensitive cultivars under different salinity levels, suggesting that the significant differences observed in activities of NR, GS and GPT might contribute to the different Bt protein expression between two cultivars.

To summarize, Bt protein content of cotton squares significantly reduced when Bt cotton plants were subjected to high salinity stress. Reduced protein synthesis, elevated protein degradation and declined ability of root absorption caused by salinity stress all resulted in the reduced Bt protein expression, and thus decreased insecticidal efficiency and final yield. Thus, optimized management strategies such as planting salt-tolerant cultivars, appropriate nitrogen fertilizer application and employing more chemical control should be applied to Bt cotton grown in saline land.

## References

[1] Wu KM, Lu YH, Feng H Q, Jiang YY, Zhao JZ. Suppression of cotton bollworm in multiple crops in china in areas with Bt toxin-containing cotton. Science. 2009; 321(5896): 1676–1678. 10.1126/science.1160550 18801998

[2] Lu Y, Wu K, Jiang Y, Guo Y, Desneux N. Widespread adoption of Bt cotton and insecticide decrease promotes biocontrol services. Nature. 2012; 487(487): 362–365. 22722864 10.1038/nature11153

[3] Lu YH, Wu KM, Jiang YY, Bing X, Ping L, Feng H Q, et al Mirid bug outbreaks in multiple crops correlated with wide-scale adoption of Bt cotton in china. Science. 2010; 328(5982): 1151–1154. 10.1126/science.1187881 20466880

[4] Benedict JH, Sachs ES, Altman DW, Deaton WR, Kohel RJ, Ring DR, et al Field performance of cottons expressing transgenic Cry1A insecticidal proteins for resistance to *Heliothis virescens* and *Helicoverpa zea* (Lepidoptera: Noctuidae). J Econ Entomol. 1996; 89(1): 230–238.

[5] Olsen KM, Daly JC. 2000 Plant-toxin interactions in transgenic Bt cotton and their effect on mortality of *Helicoverpa armigera* (Lepidoptera: Noctuidae). J Econ Entomol. 2000; 93(4): 1293–1299. 10.1603/0022-0493-93.4.1293 10985045

[6] Shen P, Lin KJ, Zhang YJ, Wu KM, Guo YY. Seasonal expression of *Bacillius thuingiensis* insecticidal protein and control to cotton bollworm in different varieties of transgenic cotton. Cotton Sci. 2010; 22: 393–397.

[7] Kumar R, Dahiya KK, Kumar D. Evaluation of Bt cotton hybrids against bollworms in cotton. Ann Agri Bio Res. 2013; 18(1): 39–43.

[8] Adamczyk JJ Jr, Sumerford DV. Potential factors impacting season-long expression of Cry1Ac in 13 commercial varieties of Bollgard cotton. J Insect Sci. 2001; 1: 1–6. 10.1673/031.001.1301 15455073 PMC355897

[9] Kranthi KR, Naidu S, Dhawad CS, Tatwawadi A, Mate K, Patil E, et al Temporal and intra-plant variability of Cry1Ac expression in Bt-cotton and its influence on the survival of the cotton bollworm, *Helicoverpa armigera* (Hubner). Curr Sci. 2005; 89: 291–298.

[10] Manjunatha R, Pradeep S, Sridhara S, Manjunatha M, Naik MI, Shivanna BK, et al Quantification of Cry1Ac protein over time in different tissues of Bt cotton hybrids. Karnataka J Agric Sci. 2009; 13(1):67–70.

[11] Stam M, Mol J N M, Kooter J M. 1997. The silence of genes in transgenic plants. Ann Bot. 1979; 79(1): 3–12.

[12] Gore J, Adamczyk JJ. Impact of bollworm, *Helicoverpa Zea* (Boddie), on maturity and yields of Bollgard and Bollgard II cottons. J Cotton Sci. 2004; 8: 223–229.

[13] Mahon R, Finnergan J, Olsen K, Lawrence L. Environmental stress and the efficacy of Bt cotton. Aust Cotton Grow. 2002; 22: 18–21.

[14] Rochester IJ. Effect of genotype, edaphic, environmental conditions, and agronomic practices on Cry1Ac protein expression in transgenic cotton. J Cotton Sci. 2006; 10(4): 252–262.

[15] Jiang LJ, Duan LS, Tian XL, Wang BM, Zhang HF, Zhang MC, et al NaCl salinity stress decreased *Bacillus thuringiensis* (Bt) protein content of transgenic Bt cotton (*Gossypium hirsutum* L.) seedlings. Environ Exp Bot. 2006; 55(3): 315–320.

[16] Iqbal A, Ali S, Zia MA, Shahzad A, Din JU, Asad MAU, et al Comparative account of Bt gene expression in cotton under normal and salt affected Soil. Int J Agric Biol. 2013; 15(6):1181–1186.

[17] Luo Z, Dong HZ, Li WJ, Ming Z, Zhu Y. Individual and combined effects of salinity and waterlogging on Cry1Ac expression and insecticidal efficacy of Bt cotton. Crop Prot. 2008; 27(12): 1485–1490.

[18] Luo JY, Zhang S, Peng J, Zhu XZ, Lv LM, Wang CY, et al Effects of soil salinity on the expression of Bt toxin (Cry1Ac) and the control efficiency of *Helicoverpa armigera* in field- grown transgenic Bt cotton. Plos One. 2017; 12(1): e0170379 10.1371/journal.pone.0170379 28099508 PMC5242435

[19] Li MY. Effect of NaCl on growth and the insect-resistance efficiency of transgenic Bt cotton (Gossypium hirsutum L.) and application plant regulator alleviate the adverse effects. Ph D thesis, China Agricultural University. 2014.

[20] Chen DH, Ye GY, Yang CQ, Chen Y, Wu YK. Effect of introducing *Bacillus thuringiensis* gene on nitrogen metabolism in cotton. Field Crops Res. 2005; 92(1): 1–9.

[21] Li Y, Li YB, Hu DP, Wang J, Heng L, Zhang X, et al Effects of waterlogging on Bt protein content and nitrogen metabolism in square of Bt cotton. Acta Agronomica Sinica. 2017; 43(11): 1658–1666.

[22] Chen Y, Wen Y, Chen Y, Cothren JT, Zhang X, Wang YH, et al Effects of extreme air temperature and humidity on the insecticidal expression level of Bt cotton. J Integr Agric. 2012; 11(11): 1836–1844.

[23] Silberbush M, Ben AJ. The effect of salinity on parameters of potassium and nitrate uptake of cotton. Commun Soil Sci Plant Anal. 1987; 18(1): 65–81.

[24] Kumari A, Das P, Parida AK, Agarwal PK. Proteomics, metabolomics, and ionomic sperspectives of salinity tolerance in halophytes. Front Plant Sci. 2015; 6: 537 10.3389/fpls.2015.00537 26284080 PMC4518276

[25] Mokhele B, Zhan X, Yang G, Zhang X. Nitrogen assimilation in crop plants and its affecting factors. Can J Plant Sci. 2015; 92(3): 399–405.

[26] Liu RX, Yang CQ, Zhang GW, Zhang L, Yang FQ, Guo WQ. Root recovery development and activity of cotton plants after waterlogging. Agron J. 2015; 107(6): 2038–2046.

[27] Zouhaier B, Mariem M, Mokded R, Rouached A, Alsane K, Chedly A, et al Physiological and biochemical responses of the forage legume *Trifolium alexandrinum* to different saline conditions and nitrogen levels. J Plant Res. 2016; 129(3): 423–434. 10.1007/s10265-016-0791-6 26818949

[28] Xin CS, Dong HZ, Wen SM, Zhang XJ, Xin MP. Salt tolerance appraisal of Bt cotton with different genotypes in coastal saline soil. Chin Agri Sci Bul. 2008; 24: 188–192.

[29] Zhang LN, Ye WW, Wang JJ, Fan BX, Wang DL. Genetic diversity analysis of salinity related germplasm in cotton. Biodivers Sci. 2010; 18(2): 142–149.

[30] Chen S, Wu JY, He XL, Huang JQ, Zhou BL, Zhang RX. Quantification using ELISA of *Bacillus thuringiensis* insecticidal protein expressed in the tissue of transgenic insect-resistant cotton. J Jiangsu Agr Sci. 1997; 3: 154–156.

[31] Weiler EW, Jourdan PS, Conrad W. Levels of indole-3-aceticacid and intact decapitated coleoptiples as determined by a specific and highly sensitive solid-phase enzyme immuno-assay. Planta. 1981; 153(6): 561–571. 10.1007/BF00385542 24275876

[32] Liu RX, Zhou ZG, Guo WQ, Chen BL, Oosterhuis DM. Effects of N fertilization on root development and activity of water-stressed cotton (*Gossypium hirsutum* L.) plants. Agric Water Manag. 2008; 95(11): 1261–1270.

[33] Kong XS, Yi XF. Techniques of plant physiological experiment Beijing: China Agriculture Press; 2008.

[34] Nelson D, Sommers L. A simple digestion procedure for estimation of total nitrogen in soils and sediments. J Environ Qual. 1972; 1(4): 423–425.

[35] Yemm EW, Cocking EC, Ricketts R. Determination of amino acids with ninhydrin. Analyst. 1955; 80: 209–214.

[36] Bradford MM. A rapid and sensitive method for the quantification of microgram quantities of protein utilizing the principle of protein-dye binding. Anal Biochem. 1976; 72: 248–254. 942051 10.1016/0003-2697(76)90527-3

[37] Ding Y, Luo W, Xu G. Characterisation of magnesium nutrition and interaction of magnesium and potassium in rice. Ann Appl Biol. 2006; 149(2): 111–123.

[38] Oaks A, Stulen I, Jones K, Winspear M J, Misra S, Boesel IL. Enzymes of nitrogen assimilation in maize roots. Planta. 1980; 148(5): 477–484. 10.1007/BF00552663 24310191

[39] Wu LH, Jiang SH, Tao QN. Plant aminotransferase (GOT and GPT) determination method and its application of activity colorimetric. Chin J Soil Sci. 1998; 29: 136–138.

[40] Tonhazy NE, White NG, Umbriet WW. Colorimetric assay of glutamic-pyruvic transaminase. Arch Biochem Biophys. 1950; 28: 36–38. 14771923

[41] Carrasco P, Carbonell J. Changes in the level of peptidase activities in pea ovaries during senescence and fruit set induced by gibberellic acid. Plant Physiol. 1990; 92(4): 1070–1074. 16667372 10.1104/pp.92.4.1070 PMC1062417

[42] Setlow P. Protease and peptidase activities in growing and sporulating cells and dormant spores of *Bacillus megaterium*. J Bacteriol. 1975; 122(2): 642–649. 805126 10.1128/jb.122.2.642-649.1975 PMC246102

[43] Vance CP, Heichel GH, Barnes DK, Bryan JW, Johnson LE. Nitrogen fixation, nodule development, and vegetative regrowth of alfalfa (*Medicago sativa* L.) following harvest. Plant Physiol. 1981; 67(6): 1198–1203. 16660893 10.1104/pp.64.1.1 PMC543014

[44] Ashraf M, Ahmad S. Influence of sodium chloride on ion accumulation, yield components and fiber characteristics in salt-tolerant and salt-sensitive lines of cotton. Field Crops Res. 2000; 66(2): 115–127.

[45] Blaise D, Kranthi KR. Cry1Ac expression in transgenic Bt cotton hybrids is influenced by soil moisture and depth. Curr. Sci. 2011; 101(6): 783–786.

[46] Martins CM, Beyene G, Hofs JL, Kruger K, Vyver CV, Schluter U, et al Effect of water-deficit stress on cotton plants expressing the *Bacillus thuringiensis* toxin. Ann Appl Biol. 2008; 152(2): 255–262.

[47] Millar AA, Dennis ES. Protein synthesis during oxygen deprivation in cotton. Funct Plant Biol. 1996; 23(23): 341–348.

[48] Khan A, Tan DKY, Afridi MZ, Luo H, Tung SA, Fahad S. Nitrogen fertility and abiotic stresses management in cotton crop: a review. Environ Sci Pollut Res Int. 2017; 24(17):14551–14566. 10.1007/s11356-017-8920-x 28434155

[49] Shah JM, Bukhari SAH, Zeng JB, Quan XY, Ali E, Muhammad N, et al Nitrogen (N) metabolism related enzyme activities, cell ultrastructure and nutrient contents as affected by N level and barley genotype. J Integr Agr. 2017, 16(1): 190–198.

[50] Ashrafa M, Shahzada SM, Imtiazb M, Rizwanb MS. Salinity effects on nitrogen metabolism in plants- focusing on the activities of nitrogen metabolizing enzymes: A review. J Plant Nutr. 2018; 41(8):1–17.

[51] Wang X, Yang P, Gao Q, Liu X, Kuang T, Shen S, et al Proteomic analysis of the response to high-salinity stress in Physcomitrella patens. Planta. 2008, 228(1):167–177. 10.1007/s00425-008-0727-z 18351383

[52] Meng S, Zhang C, Sua L, Lia Y, Zhao Z. Nitrogen uptake and metabolism of Populus simonii in response to PEG-induced drought stress. Environ Exp Bot. 2016; 123: 78–87.

[53] Siddiqui MH, Mohammad F, Khan MN, Alwhaibi MH, Bahkali AHA. Nitrogen in relation to photosynthetic capacity and accumulation of osmoprotectant and nutrients in *Brassica* genotypes grown under salt stress. Agr Sci China. 2010; 9(5): 671–680.

[54] Zhang X, Wang J, Peng P, Li Y, Tian X, Wang G, et al Effects of soil water Deficit on insecticidal protein expression in boll shells of transgenic Bt cotton and the mechanism. Front Plant Sci. 2017; 8 10.1016/S1671-2927(09)60142-5 PMC5732147 29321788

[55] Miller A, Cramer M. Root nitrogen acquisition and assimilation. Plant Soil. 2005; 274: 1–36.

[56] Chen W, Hou Z, Wu L, Liang Y, Wei C. Effects of salinity and nitrogen on cotton growth in arid environment. Plant Soil. 2010; 326(1):61–73.

[57] Higbie SM, Wang F, Stewart JM, Sterling TM, Lindemann WC, Hughs E, et al Physiological response to salt (NaCl) stress in selected cultivated tetraploid cottons. Int J Agron. 2010; 2010:1–12.

[58] Zhang HJ, Dong HZ, Li WJ, Zhang DM. Effects of soil salinity and plant density on yield and leaf senescence of field-grown cotton. J Agron Crop Sci. 2012; 198(1):27–37.

[59] Xu Y, Burgess P, Huang B. Root antioxidant mechanisms in relation to root thermotolerance in perennial grass species contrasting in heat tolerance. Plos One. 2015; 10(9): e0138268 10.1371/journal.pone.0138268 26382960 PMC4575078

[60] Khan TA, Mazid M, Mohammad FA. Review of ascorbic acid potentialities against oxidative stress induced in plants. J Agro boil. 2011; 28:97–111.

[61] Zhang L, Ma H, Chen T, Pen J, Yu S, Zhao X. Morphological and physiological responses of cotton (*Gossypium hirsutum* L.) plants to salinity. Plos One. 2014; 9(11): e112807 10.1371/journal.pone.0112807 25391141 PMC4229235

[62] Li M, Li F, Yue Y, Tian X, Li Z, Duan L. NaCl-induced changes of ion fluxes in roots of transgenic *Bacillus thuringiensis* (Bt) cotton. J Integr Agr. 2013; 12(3): 436–444.

